# PKCα Binds G3BP2 and Regulates Stress Granule Formation Following Cellular Stress

**DOI:** 10.1371/journal.pone.0035820

**Published:** 2012-04-20

**Authors:** Tamae Kobayashi, Sofia Winslow, Lovisa Sunesson, Ulf Hellman, Christer Larsson

**Affiliations:** 1 Center for Molecular Pathology, Lund University, Malmö, Sweden; 2 Ludwig Institute for Cancer Research Ltd., Uppsala University, Uppsala, Sweden; Hertie Institute for Clinical Brain Research and German Center for Neurodegenerative Diseases, Germany

## Abstract

Protein kinase C (PKC) isoforms regulate a number of processes crucial for the fate of a cell. In this study we identify previously unrecognized interaction partners of PKCα and a novel role for PKCα in the regulation of stress granule formation during cellular stress. Three RNA-binding proteins, cytoplasmic poly(A)^+^ binding protein (PABPC1), IGF-II mRNA binding protein 3 (IGF2BP3), and RasGAP binding protein 2 (G3BP2) all co-precipitate with PKCα. RNase treatment abolished the association with IGF2BP3 and PABPC1 whereas the PKCα-G3BP2 interaction was largely resistant to this. Furthermore, interactions between recombinant PKCα and G3BP2 indicated that the interaction is direct and PKCα can phosphorylate G3BP2 *in vitro*. The binding is mediated via the regulatory domain of PKCα and the C-terminal RNA-binding domain of G3BP2. Both proteins relocate to and co-localize in stress granules, but not to P-bodies, when cells are subjected to stress. Heat shock-induced stress granule assembly and phosphorylation of eIF2α are suppressed following downregulation of PKCα by siRNA. In conclusion this study identifies novel interaction partners of PKCα and a novel role for PKCα in regulation of stress granules.

## Introduction

Protein kinase C (PKC) is a family of serine/threonine kinases that play important roles in several processes that control cell fate such as apoptosis, proliferation, and differentiation. The PKC isoforms are grouped in classical (PKCα, βI, βII, and γ), novel (PKCδ, ε, η, and θ) and atypical (PKCζ, and ι) PKCs [1]. Each isoform can be specifically regulated and has unique functions in a given cell. This is conceivably in part attributed to differential sensitivities to activating factors that are increased upon stimulation of cell surface receptors. For example, only classical isoforms are activated by Ca^2+^ and only classical and novel isoforms are sensitive to diacylglycerol. In addition to these differences in activator sensitivity, a wide range of studies indicate that isoform-specific interactions with other proteins largely determine the function of a PKC isoform [2].

In order to further understand PKC isoform function we have screened for interaction partners by immunoprecipitating a neuritogenic PKCε structure and identified co-precipitated proteins by mass spectrometry analysis. One of these was the intermediate filament protein peripherin [3]. In addition, several proteins containing RNA recognition motifs (RRMs) were identified. These include the cytoplasmic poly(A)^+^ binding protein (PABPC1), the IGF-II mRNA binding protein 3 (IGF2BP3), and the RasGAP binding protein 2 (G3BP2).

PABPC1 binds to the poly(A) tail of mRNAs and its primary role is conceivably in regulating translation initiation and mRNA stability [4]. IGF2BP3 was first detected overexpressed in pancreatic cancer and initially called KOC (after *K*H domain containing protein *o*verexpressed in *c*ancer) but later identified as an IGF-II mRNA-binding protein and re-named [5], [6]. It is one of three members of the IGF-II mRNA-binding protein (IMP) family which are important for transporting their mRNA targets to proper cellular localization during development [7]. G3BP2 and the closely related protein G3BP1, which has been more studied, may have several roles in the cell. They have both been shown to interact with SH3 domains in GTPase-activating proteins [8], [9] and with MYC mRNA [10] regulating its turnover. Furthermore, overexpression of G3BP1 leads to the assembly of stress granules which sequester and retain mRNA-species that are not supposed to be translated during the cellular stress response [11], [12].

When analyzing endogenous proteins we could detect an interaction between PKCα and the three mRNA-binding proteins. The fact that all proteins contain RNA-binding domains, and that at least PABPC1 and G3BP are known to localize to stress granules, led us to raise the hypothesis that PKC may participate in stress granule formation. In this paper we demonstrate that PKCα both regulates the assembly of stress granules and is a component of them. The results therefore provide information regarding novel cellular roles of PKCα.

## Results

### PKCα interacts with G3BP2

**Figure 1:**
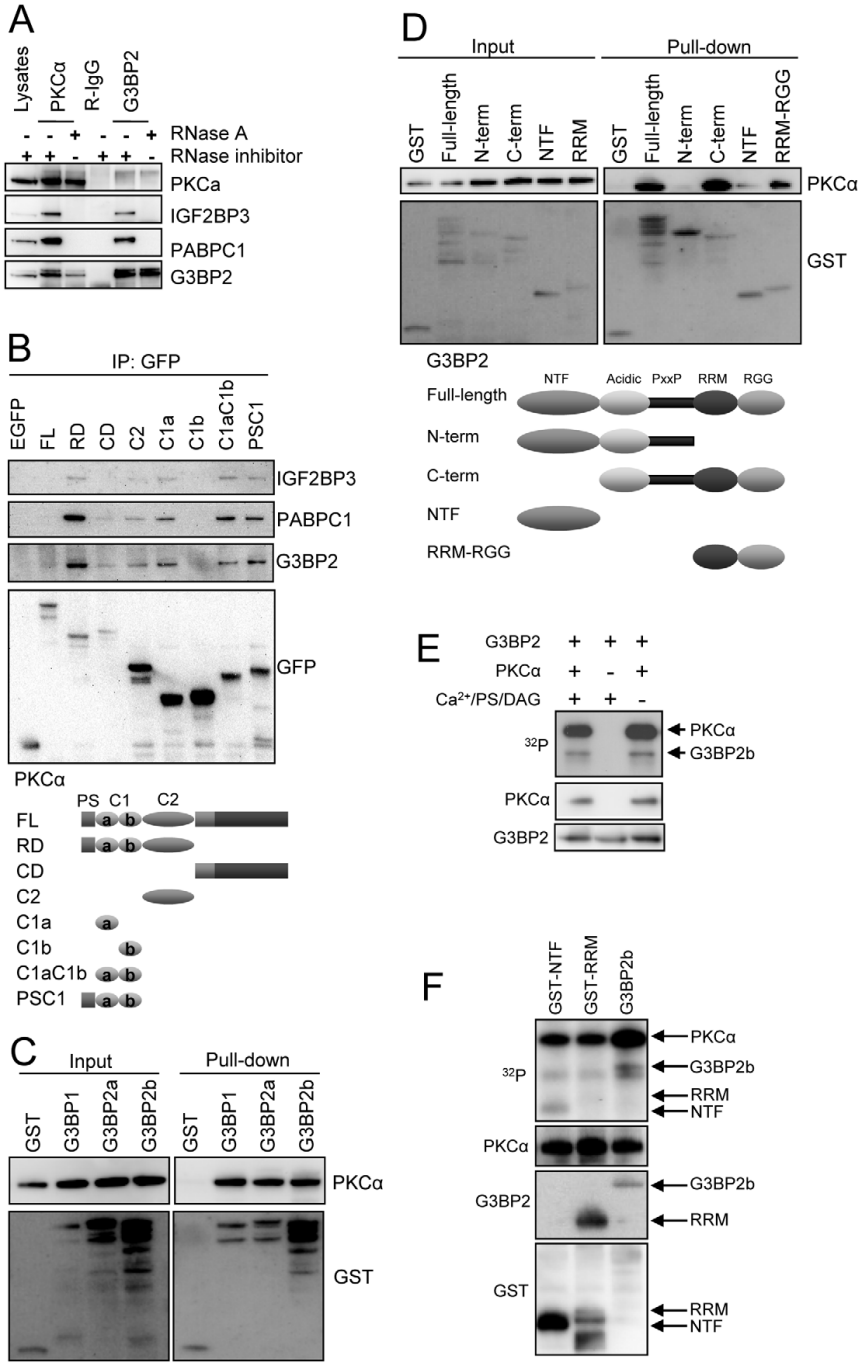
Interaction of PKCα with G3BP2, IGF2BP3 and PABPC1. (A) PKCα and G3BP2 were immunoprecipitated from SK-N-BE(2)C cell lysates that had been treated with RNase or a RNase inhibitor. Immunoprecipitates were thereafter subjected to Western blot with indicated antibodies. (B) SK-N-BE(2)C cells were transfected with vectors encoding EGFP fusions of full-length PKCα (FL) or the regulatory (RD), catalytic (CD), C2, C1a, C1b, tandem C1a and C1b (C1ab), or PSC1 (PSC1) domains. The constructs are illustrated below the blot. Cell lysates were immunoprecipitated using anti-GFP-conjugated magnetic beads. Lysates and precipitates were thereafter analyzed with Western blot using IGF2BP3, PABPC1, G3BP2 or GFP antibodies. PKCα was incubated with GST-tagged G3BP1, G3BP2a and G3BP2b (C) or different G3BP2b constructs (D). Thereafter GST was pulled down with GSH-coupled sepharose. Incubation mixture and pull-downs were analyzed with Western blot. Schematic illustration of the G3BP2 constructs is shown below the blot. (E) G3BP2b (with GST cleaved off) was incubated with PKCα, [γ-^32^P]ATP and PKC activators (Ca^2+^/PS/DAG - Ca^2+^, phosphatidylserine and diacylglycerol) as indicated. The reactions were separated on SDS-PAGE, blotted and [^32^P] was visualized with autoradiography and proteins with immunoblotting. (F) G3BP2b (with GST cleaved off) and GST-tagged G3BP2 domains were incubated with PKCα, [γ-^32^P]ATP and PKC activators. The reactions were analyzed as in (E).

In a screen for proteins that interact with an overexpressed PKCε construct (PKCεPSC1V3) [3], mass spectrometry analysis of some of the co-precipitated proteins resulted in the identification of the RNA-binding proteins IGF2BP3, PABPC1, and G3BP2. Although the interactions could be confirmed for overexpressed PKCε constructs we could not detect endogenous interactions under normal growth conditions. However, we found that the proteins interacted with PKCα (Fig. 1).

The fact that the identified PKC-interacting proteins also are RNA-binding proteins raised the possibility that the interaction is dependent on intact RNA. We therefore analysed the interaction in cell lysates that had been treated with either RNase inhibitor or RNase prior to immunoprecipitation (Fig. 1A). The interaction of PKCα with IGF2BP3 and PABPC1 was abolished by RNase treatment whereas G3BP2 and PKCα could still be co-precipitated with each other. G3BP2 itself interacted with IGF2BP3 and PABPC1 in an RNA-dependent manner, which could suggest that the proteins are in a common complex.

To analyze the structures in PKCα that mediate the interactions, EGFP-fusions of PKCα domains were expressed in SK-N-BE(2)C neuroblastoma cells and immunoprecipitated using the EGFP tag (Fig. 1B). G3BP2, PABPC1 and IGF2BP3 all co-precipitated with essentially the same PKCα constructs. The binding seems to be mediated primarily by the regulatory domain, where the C1a and C2 domains both have binding capability. On the other hand, the C1b domain did not interact with the proteins.

The data in Figure 1A indicate that there may be a direct binding between PKCα and G3BP2, whereas the association with IGF2BP3 and PABPC1 is indirect and dependent on RNA. Therefore we further analyzed the PKCα-G3BP2 interaction with a GST pull-down experiment with recombinant proteins (Fig. 1C). PKCα was pulled down together with GST-G3BP2 demonstrating a direct interaction between the proteins. Both G3BP2 variants (G3BP2a and G3BP2b which are generated by alternative splicing) bind PKCα. The binding is not specific for G3BP2 since the closely related protein G3BP1 also pulled down PKCα (Fig. 1C).

To examine which structures in G3BP2 that mediate the binding, GST-tagged domains of G3BP2 were included in the binding assay (Fig. 1D). The isolated RNA-binding domain pulled down PKCα whereas G3BP2 variants lacking this domain did not interact.

Taken together, the data suggest that the C1a and C2 domains in the regulatory domain of PKCα contain structures that can mediate the interaction with the C-terminal RNA binding domain of G3BP2.

We also analyzed if G3BP2 is a PKC substrate *in vitro* (Fig. 1E and 1F). G3BP2 was phosphorylated in the presence of PKCα, although not to the same extent as PKCα itself. The phosphorylation took place also in the absence of PKC activators (Fig. 1E). It is conceivable that the NTF, but not the RRM, domain contains PKC phosphorylation sites since GST-NTF but not GST-RRM was phosphorylated in the kinase assay (Fig. 1F).

### A slower migrating PKCα variant is enriched in G3BP2 precipitates

**Figure 2:**
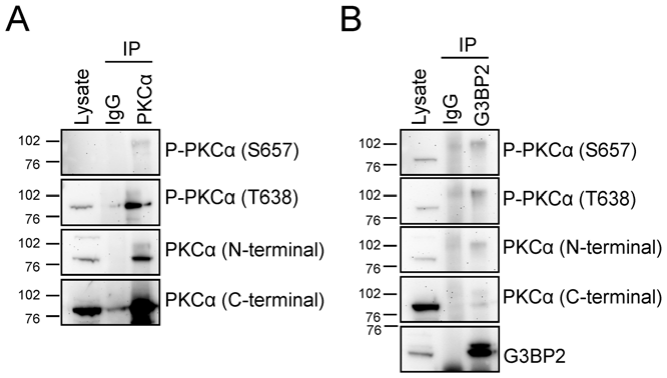
Analysis of the PKCα variant co-precipitated with G3BP2. Lysates from SK-N-BE(2)C neuroblastoma cells were incubated with either PKCα antibody (A) or G3BP2 antibody (B) and precipitated materials were separated by SDS-PAGE. Co-immunoprecipitated proteins were visualized with Western blot using antibodies indicated in the figure. P-PKCα, Phosphorylated PKCα.

As can be seen in Figure 1A the major PKCα species that is co-immunoprecipitated with G3BP2 does not migrate as fast as the major PKCα species in the PKCα immunoprecipitate. To certify that this is PKCα and to investigate whether there is a difference in post-translational modifications of the PKCα variants, PKCα and G3BP2 immunoprecipitates were probed with different PKCα antibodies (Fig. 2). The upper band, enriched in G3BP2 precipitates, is clearly recognized by an antibody towards the N-terminal region of PKCα but more weakly by an antibody towards the C-terminal region. The antibody recognizing phosphorylated T638 identified both PKCα bands in a ratio similar as the general PKCα antibodies (Fig. 2A). However an antibody directed towards phosphorylated S657 primarily reacted with the upper band. Both antibodies towards phosphorylated PKC recognized the PKCα found in G3BP2 precipitates (Fig. 2B). Thus, the fact that several PKCα antibodies react with the slow-migrating species in the G3BP2 precipitate underscores that it is PKCα. G3BP2 primarily interacts with a PKCα variant that has both its C-terminal phosphorylation sites phosphorylated.

### PKCα as well as G3BP2, IGF2BP3 and PABPC1 localizes to stress granules

**Figure 3:**
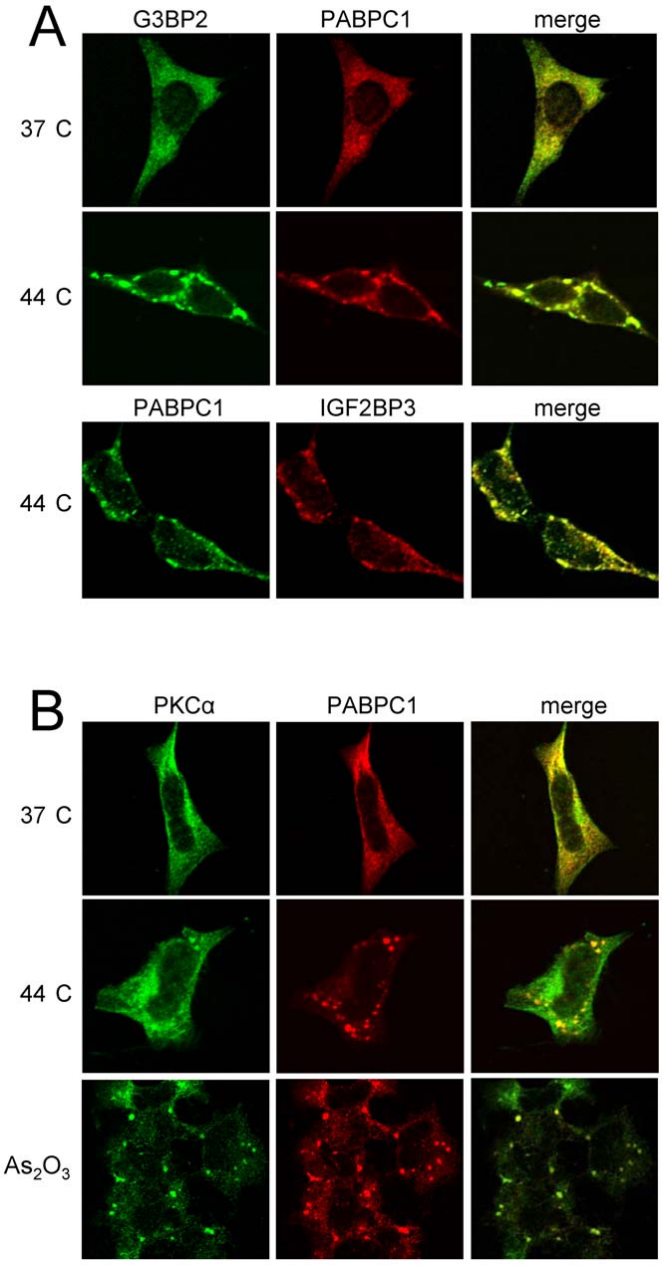
PKCα, G3BP2, PABPC1 and IGF2BP3 co-localize in stress granules. (A) SK-N-BE(2)C cells were either incubated at 37°C or heat shocked at 44°C for 1 h and endogenous G3BP2, IGF2BP3, and PABPC1 were visualized by immunofluorescence. (B) Cells subjected to heat shock or treated with 600 µM of As_2_O_3_ for 1 h were analyzed with immunofluorescence towards PKCα. PAPBC1 was used as stress granule marker. Cells were examined with confocal microscopy.

PABPC1 and G3BP1 (closely related to G3BP2 and often referred to as G3BP) have previously been shown to be associated with the formation of stress granules during cellular stress [11], [13]. We therefore investigated whether the three PKC-interacting proteins co-localize during stress. SK-N-BE(2)C cells were incubated at 44°C and PABPC1, G3BP2 and IGF2BP3 proteins were visualized by immunofluorescence (Fig. 3A). Under normal conditions PABPC1, G3BP2 and IGF2BP3 (not shown) are diffusely localized in the cytosol, whereas upon heat shock the proteins relocate to newly formed stress granules. Thus, the proteins co-localize in stress granules under these conditions.

**Figure 4:**
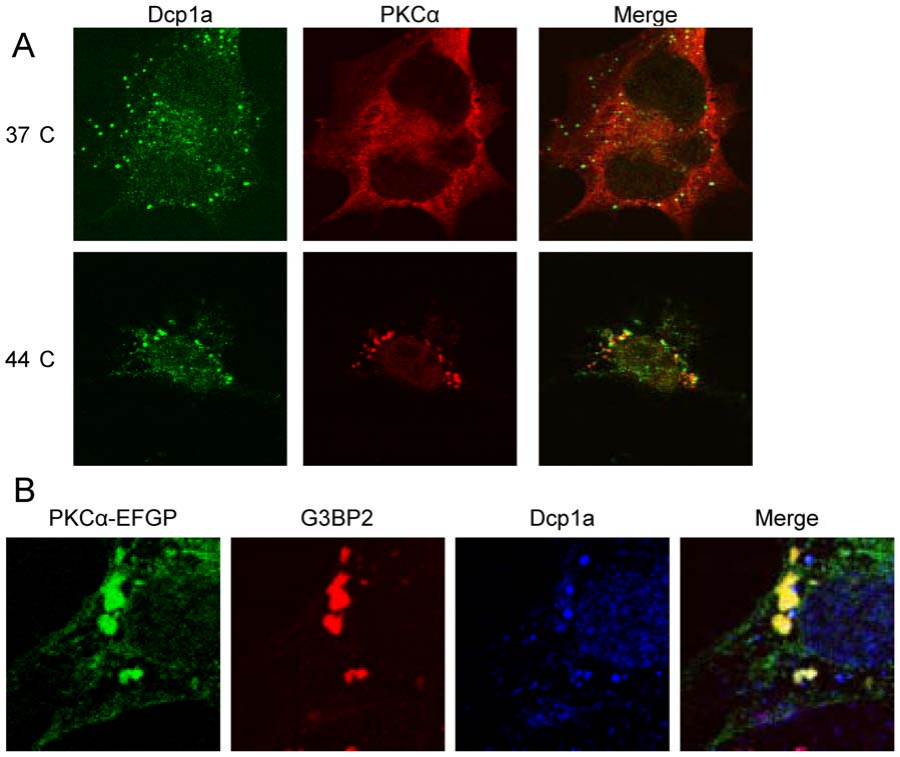
PKCα does not localize to P-bodies. Non-treated SK-N-BE(2)C cells (A) or SK-N-BE(2)C cells transfected with a vector encoding PKCα-EGFP (B) were placed at 44°C for 1 h. PKCα and the P-body marker Dcp1a (A) or Dcp1a and G3BP2 (B) were thereafter visualized by immunofluorescence. Cells were analyzed by confocal microscopy and in (B) PKCα was detected by the EGFP fluorescence.

To investigate whether PKCα accompanies the identified mRNA-binding proteins to stress granules SK-N-BE(2)C cells were subjected to heat shock and PKCα and PABPC1 were visualized by immunofluorescence (Fig. 3B). PKCα and PABPC1 both showed a diffuse cytosolic localization pattern in cells cultured at 37°C. After 1 h of heat shock PABPC1-containing stress granules were formed and PKCα was present in many of the PABPC1-containing granules (Fig. 3B). Accumulation of PKCα in PABPC1-containing stress granules was also observed after treatment with As_2_O_3_ (Fig. 3B) indicating that the PKCα relocation is not limited to the stress response induced by heat shock. A weak increase of PKCα reactivity in the nucleus could be seen after heat shock (Figs. 3B and 4A) but this was not the case following As_2_O_3_ treatment.

Processing-bodies (P-bodies) constitute another class of mRNA-rich granules that are functionally and spatially linked to stress granules [14], [15]. To analyze whether PKCα also localizes to these structures, SK-N-BE(2)C cells were subjected to heat shock. PKCα and the P-body marker Dcp1a were thereafter visualized with immunofluorescence (Fig. 4A). PKCα could not be detected in Dcp1a-positive structures.

To simultaneously visualize P-bodies, stress granules and PKCα, the cells were transfected with a vector encoding EGFP-tagged PKCα and stained for G3BP2 and Dcp1a (Fig. 4B). Stress granules and P-bodies were in many cases localized immediately adjacent to each other with some overlapping pixels in the borders of the structures. However, PKCα displayed a clear co-localization with stress granules whereas isolated P-bodies were PKCα-negative.

### The PKCαC1a domain associates with stress granule proteins after heat shock

**Figure 5:**
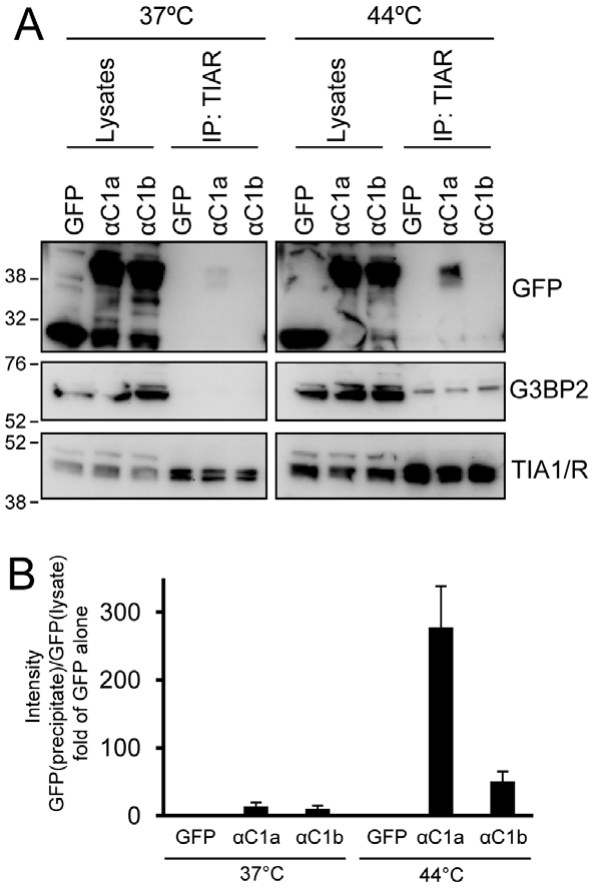
The PKCαC1a but not C1b domain is recruited to TIAR-containing complexes upon heat shock. SK-N-BE(2)C neuroblastoma cells were transfected with vectors encoding EGFP alone, or EGFP fused to the PKCα C1a or C1b domain and incubated at 37°C or 44°C for 1 h before harvesting. Cell lysates were sonicated and immunoprecipitated using anti-TIAR. Lysates and precipitates were analyzed with Western blot using antibodies indicated in the figure (A). The intensities of the EGFP fusions were quantified and the ratio of the intensity in precipate to the intensity in the lysates is shown in (B). Data are mean ± SEM, n = 3.

Our data indicate that the C1a but not the C1b domain of PKCα contains structures that can mediate its interaction with G3BP2 (Fig. 1B). To investigate if it also can mediate the association with stress granule components, the PKCαC1a and PKCαC1b domains were expressed in SK-N-BE(2)C cells that were subsequently subjected to heat shock. To enrich for stress granule components we immunoprecipitated the stress granule component TIAR (Fig. 5). As expected, G3BP2 was co-precipitated with TIAR following heat shock, indicating that stress granule components are enriched in the precipitate. The experiment revealed that the PKCαC1a domain, but not the PKCαC1b domain, co-precipitates with TIAR upon heat shock, indicating that the C1a domain can mediate interaction with stress granule components.

### Downregulation of PKCα delays stress granule formation

**Figure 6:**
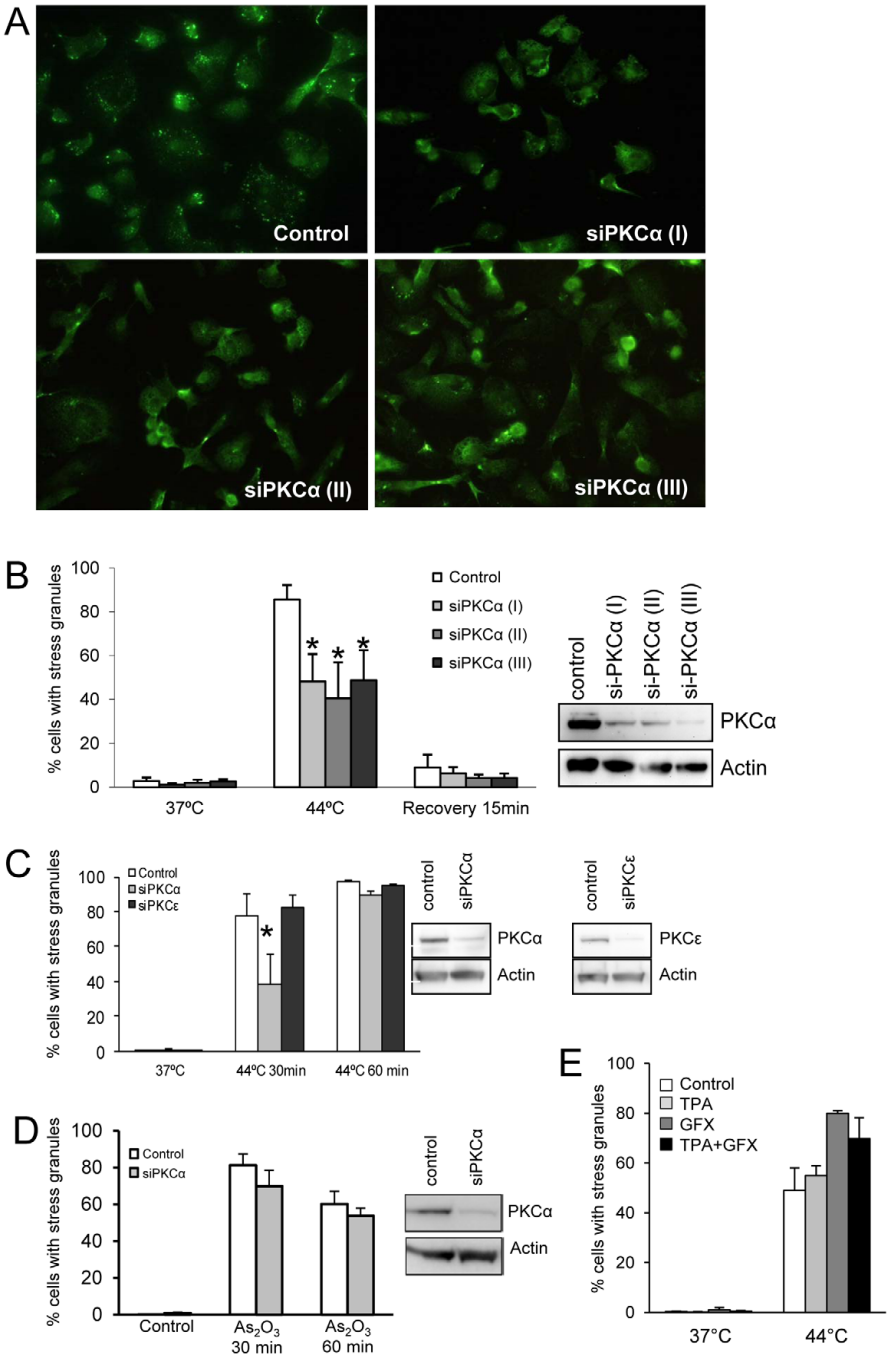
Downregulation of PKCα suppresses stress granule assembly. MDA-MB-231 cells were transfected with three different siRNA oligonucleotides against PKCα, and were thereafter subjected to heat shock at 44°C for 1 h. Stress granules were visualized with immunofluorescence using a G3BP2 antibody (A). Western blot of cell lysates demonstrating PKCα downregulation and quantification of the percentage of cells containing stress granules (B) (mean ± SEM, n = 3). (C) MDA-MB-231 cells were treated with siRNAs targeting PKCα or PKCε, followed by heat shock for indicated time periods. Cell lysates were analyzed with Western blot demonstrating downregulation of respective isoforms. Stress granules were visualized by G3BP2 immunofluorescence and the percentage of cells with stress granules was quantified (mean ± SEM, n = 3). (D) MDA-MB-231 cells with downregulated PKCα were treated with 300 µM As_2_O_3_ for 30 or 60 minutes. Cell lysates were analyzed with Western blot and the percentage of cells with stress granules, identified by PABPC1 immunofluorescence was quantified (mean ± SEM, n = 3). (E) MDA-MB-231 cells were treated with 16 nM TPA and/or 2 µM GF109203X (GFX) during heat shock. Stress granule-positive cells were thereafter quantified. * denotes statistically significant (p<0.05) difference compared to control using ANOVA followed by Duncan's multiple range test.

Since PKCα interacts with stress granule components and also localizes to these structures we postulated that PKCα may be involved in the regulation of stress granule formation. To test this hypothesis we aimed at downregulating PKCα with siRNA and study the stress granule induction. Due to difficulties in obtaining substantial knockdown of PKCα in SK-N-BE(2)C cells, we used the breast carcinoma MDA-MB-231 cell line for these experiments. PKCα localizes to stress granules upon heat shock also in MDA-MB-231 cells (data not shown). MDA-MB-231 cells were transfected with three different PKCα siRNA oligonucleotides and were thereafter subjected to a 44°C heat shock (Fig. 6A and 6B). Western blot demonstrated decreased PKCα levels following transfections with PKCα siRNA. Silencing of PKCα led to a decrease of the amount of cells with stress granules under heat shock (from 86%±7% for control to 48%±13 for siPKCα(I)-, 41%±16% for siPKCα(II)-, and 49%±14% for siPKCα(III)-transfected cells; Fig. 6A and 6B). PKCα downregulation did not delay the disassembly of the stress granules following a reversal of the temperature to 37°C.

The effects of PKCα downregulation on stress granules formation was compared with knock-down of PKCε (Fig. 6C). Suppression of PKCε levels did not influence the formation of stress granules as PKCα did. PKCα downregulation only suppressed stress granule formation during the initial phase suggesting that absence of PKCα does not abolish stress granules but rather delays their assembly. The As_2_O_3_-induced stress granule assembly was not siginificantly reduced in PKCα-downregulated cells, suggesting that the importance of PKCα depends on the stress inducer (Fig. 6D).

To analyze whether PKC activity affects stress granule formation we treated cells with the PKC activator 12-*O*-tetradecanoylphorbol-13-acetate (TPA) and/or the inhibitor GF109203X concomitantly with heat shock (Fig. 6E). Neither agent induced stress granules by themselves. However, the heat shock-induced stress granule formation was potentiated by the PKC inhibitor.

### Downregulation of PKCα delays heat shock-induced phosphorylation of eIF2α

**Figure 7:**
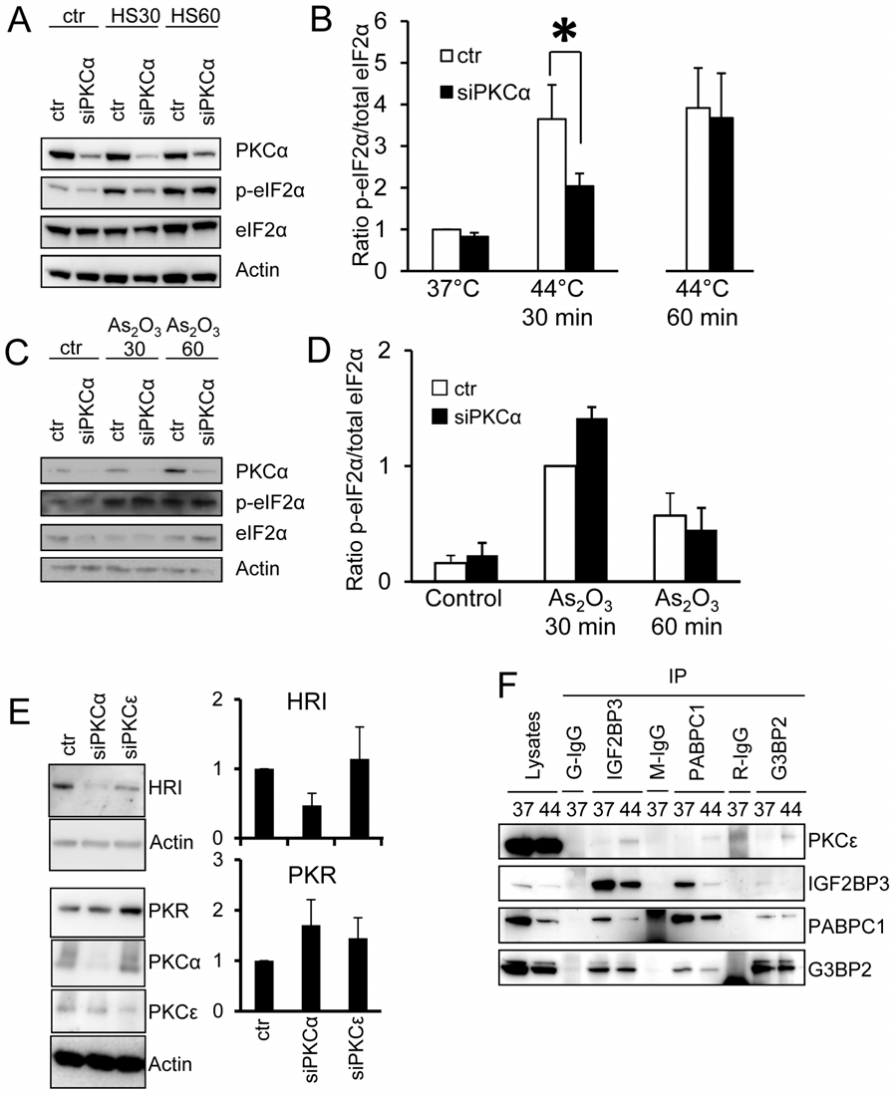
Heat shock-induced phosphorylation of eIF2α is delayed in cells with downregulated PKCα. PKCα was downregulated in MDA-MB-231 cells by siRNA prior to subjection to heat shock (A and B) or As_2_O_3_ treatment (C and D) for indicated time periods. Lysates were analyzed for phosphorylated eIF2α, total eIF2α, PKCα and actin by Western blot (A and C). The levels were quantified and related to total eIF2α and normalized to values obtained in control cells treated with a control siRNA (B and D) (mean ± SEM, n = 3). (E) PKCα and PKCε were downregulated in MDA-MB-231 cells and the expression levels of HRI and PKR were analyzed with Western blot. The graphs show quantification of HRI and PKR levels divided by actin levels and normalized to control. Data are mean ± SEM, n = 4. (F) Lysates from cells that had or had not been subjected to heat shock were immunoprecipitated with antibodies towards IGF2BP3, PABPC1 and G3BP2 or matching isotype controls. The precipitates were thereafter analyzed for the presence of PKCε. * denotes statistically significant (p<0.05) difference compared to control using ANOVA followed by Duncan's multiple range test.

Translational arrest by phosphorylation of eukaryotic translation initiation factor 2α (eIF2α) is one of the major triggers that induce stress granule formation. We therefore analyzed if down-regulation of PKCα also leads to suppression of the heat shock-induced eIF2α phosphorylation (Fig. 7A and 7B). As for stress granule formation, there was a suppression of the initial phosphorylation of eIF2α whereas after prolonged stress no effect of PKCα downregulation could be discerned. As_2_O_3_ exposure also leads to increased phosphorylation of eIF2α. However, contrary to heat shock, we could not detect a suppression of the phosphorylation in PKCα-depleted cells (Fig. 7C and 7D).

To investigate whether downregulation of a kinase upstream of eIF2α could explain the effect of PKCα depletion on heat shock-induced eIF2α phosphorylation we analyzed the levels of protein kinase R (PKR) and heme-regulated inhibitor kinase (HRI), two kinases that may mediate eIF2α phosphorylation during heat shock (Fig. 7E). PKR levels were, if anything, increased in PKCα-depleted cells. However, HRI levels were lower in PKCα-depleted cell in all four experiments but the p-value was not below 0.05. As a comparison we included lysates from PKCε-depleted cells. The levels of HRI and PKR were not influenced as much in these cells.

To obtain further insights into PKCε we analyzed whether PKCε co-precipitates with IGF2BP3, PABPC1 and G3BP2 (Fig. 7F). We could not detect PKCε in either precipitate under normal growth conditions. However, following heat shock, PKCε could be discerned in IGF2BP3- and G3BP2 precipitates.

## Discussion

Here we report for the first time that PKCα is a component of stress granules and that it associates with RNA-binding proteins G3BP2, IGF2BP3 and PABPC1. These findings provide novel information regarding PKC-mediated regulation of the cellular response to stress.

Since the identified PKCα interaction partners all are RNA-binding proteins it suggests a role for PKCα in RNA regulation. Indeed the interaction with IGF2BP3 and PABPC1 was dependent on intact RNA indicating the central role of RNA for the association. On the other hand, the interaction with G3BP2 was largely resistant to RNase treatment and could also be obtained with isolated proteins *in vitro*, indicating that it is a direct binding.

It has long been recognized that PKC can influence protein synthesis by acting at the RNA level. Several studies have demonstrated that PKC activation leads to increased stability of mRNA species [16]–[21]. The mechanisms by which PKC achieves this are still largely unknown but the mRNA-binding Hu proteins are one group of potential mediators. PKC regulates the Hu proteins both by increasing their expression levels [22] with subsequent stabilization of target mRNAs and by phosphorylation which influences its shuttling in and out of the nucleus [23], [24]. A role for PKC in mRNA regulation is also supported by the identification of PKCβII in messenger ribonucleoprotein complexes [25]. Upon activation, PKCβII binds RACK1 in the complex and RACK1 was shown to bind both PABPC1 and G3BP2.

The interaction with RACK1 is also important for PKC to modulate ribosomal subunit joining [26]. Our results show that PKCα interacts with the mRNA binding proteins in cells under basal conditions, and in the case of G3BP2 the interaction is direct, further highlighting that PKCα may have a role in post-transcriptional regulation in general.

Our data particularly support a role for PKCα in the regulation of mRNA that takes place during stress. When cells are exposed to stress, translation is shifted towards synthesis of proteins of importance for the cellular stress response. The translation of other mRNAs is temporarily silenced and they accumulate in stress granules, which contain the small ribosomal subunits, translation initiation factors, and a vast array of RNA-binding proteins [12], [27]. The granules are dynamic and as soon as the cell is no longer exposed to stress stimuli they dissolve and protein translation is resumed [13]. Stress granules are formed when stress-sensitive serine/threonine kinases recognize and phosphorylate eIF2α, an important component of the translation initiation complex [28]. Alternatively formation of stress granules can be triggered when translation initiation is blocked at other steps such as inhibition of eIF4 or eIF4G activities or 80S ribosome assembly [29]–[31]. Stress granules can also be induced by overexpression of stress granule components, such as G3BP1 [11], T-cell intracellular antigen-1/T-cell intracellular antigen-related proteins (TIA-1/TIAR) [32], survival of motor neurons protein (SMN) [33], cytoplasmic polyadenylation-binding protein (CPEB) [34] and fragile X mental retardation protein (FMRP/FXR1) [35] in the absence of stress.

It is also becoming increasingly clear that stress granule assembly and disassembly are under the control of a diverse set of proteins that are not directly RNA-binding. Some act on modulating the post-translational modification of stress granule-related proteins. For example, stress granules are positive for ubiquitin [36] which apparently is of crucial importance since proteasome inhibition leads to stress granule formation [37] and the downregulation of the ubiquitin-binding protein HDAC6 suppresses their formation [36]. A functional screen revealed *O*-linked *N*-acetylglucosamine-modified proteins were enriched in stress granules and important for stress granule formation [38]. Phosphorylation of stress granule components also regulates the granules. Focal adhesion kinase (FAK)-mediated phosphorylation of Grb7 leads to its release from stress granules and is accompanied by stress granule disassembly [39] and phosphorylation of G3BP1 suppresses stress granule assembly by inhibiting its oligomerization [11]. Furthermore, the formation is microtubule-dependent [40] and potentiated following knockdown of apoptosis-inducing factors [41].

Our results, that PKCα relocates to these granules during stress and that knockdown of PKCα in MDA-MB-231 cells affects granule assembly after heat shock add PKCα to the list of stress granule regulators. PKCα depletion primarily led to a delay in the assembly which is analogous to the effect caused by depletion of importin α1 or by interference with microtubules [42]. PKCα did not localize to P-bodies demonstrating that it is not associated with all mRNA containing granules but is more specifically involved in stress granule dynamics. The fact that simultaneous incubation with a PKC inhibitor did not suppress stress granule formation indicates that PKCα kinase activity is not directly involved in the pathway leading to stress granules. PKC isoforms have in other system been shown to exert effects independently of its kinase activity [43], [44] and this may be another process regulated by PKC in a similar manner. Another alternative explanation could be that lower PKCα amounts during a longer time period may alter levels or functions of components important for stress granule formation. It is conceivable that the effects of PKCα depletion at least partially can be explained by alterations upstream of eIF2α phosphorylation since this event was also delayed in PKCα-downregulated cells. It is possible that lower levels of the upstream eIF2α kinase HRI is responsible for the suppressed heat shock-induced phosphorylation of eIF2α in PKCα-depleted cells.

It is likely that the PKCαC1a domain is one mediator of the interaction since this domain, as opposed to the structurally similar C1b domain, was associated with the RNA-binding proteins and was enriched in TIAR precipitates after heat shock. This supports an interrelation between the PKCα interaction with G3BP2 and its localization to and role in stress granule formation. The C1 domains were originally identified as the binding sites for phorbol esters [45] but a number of studies have emerged showing they can mediate protein interactions that are both PKC isoform-specific [46], [47] as well as common for several isoforms or C1 domain-containing proteins [48]–[50].

We could also see that G3BP2 preferably associates with a PKCα variant with a slower migration pattern, suggesting that, post-translational modification of PKCα is of importance for its association with G3BP2. Our analyses indicate differences in the phosphorylation pattern between the major PKCα variant and the one that is enriched in G3BP2 precipitates. One putative explanation to this difference is that a special conformation of PKCα is favorable for the interaction with G3BP2.

In conclusion the data demonstrate novel PKCα interaction partners which open up for mechanistic explanations of PKCα effects on RNA metabolism and stress granule-mediated regulation of the cellular response to stress.

## Materials and Methods

### Plasmids, antibodies and siRNA oligonucleotides

Expression vectors encoding full-length or isolated domains of human PKCα fused to enhanced green fluorescent protein (EGFP) have been described previously [43], [51], [52]. G3BP2b, G3BP2a and G3BP1 vectors were constructed by PCR of full-length templates (originally obtained from RZPD Deutsches Ressourcezentrum für Genomforschung GmbH for G3BP2b and cDNA from MDA-MB-231 cells for G3BP2a and G3BP1) introducing restriction enzyme sites adapted for cloning in pET41b vector. Primers used are listed in Table 1. All constructs were sequenced.

**Table 1:** Sequence of oligonucleotides.

siRNA oligonucleotide	sequence
control 44% GC	GACAGUUGAACGUCGAUUUGCAUUG
control 48% GC	UUACGGAUCGACUUAAGCCGUUGCA
PKCα I	CCGAGUGAAACUCACGGACUUCAAU
PKCα II	CCAUCGGAUUGUUCUUUCUUCAUAA
PKCα III	UCCAAACGGGCUUUCAGAUCCUUAU

Rabbit polyclonal antibodies towards G3BP2 were generated by Agrisera (Vännäs, Sweden). The C-terminal polypeptide (CRGTGQMEGRFTGQRR) was used as immunogen and the resulting serum was affinity purified. GST, IGF2BP3, PABPC1, TIA1/TIAR (H-120), TIAR(C-18), HRI (S-16), and PKCα (antigen in C-terminus) antibodies were obtained from Santa Cruz, PKCα rabbit monoclonal antibody (antigen in N-terminus) from Epitomics, TIA1 and PKR from Abcam, phospho-PKCα/βII (pThr638/641), phospho-PKC (pan) (pSer660 - PKCβII), and eIF2α antibody from Cell Signaling, phospho-eIF2α (pSer52) antibody from Stressgen and Cell Signaling, GFP antibody from Zymed, GST antibody from Oncogene, and Dcp1a antibody from ABNOVA. Secondary horseradish peroxidase-conjugated antibodies were obtained from Amersham Biosciences and antibodies conjugated to Alexa dyes from Molecular Probes. Control IgG was from Santa Cruz (goat IgG), Jackson ImmunoResearch (rabbit IgG) and ImmunoKontact (mouse IgG1).

Sequences of Stealth™ siRNA oligonucleotides (Invitrogen) are listed in Table 1.

### Cell culture, transfections, and stress treatments

SK-N-BE(2)C cells were grown in minimum essential medium (Sigma), whereas MDA-MB-231 cells were grown in RPMI 1640 medium (Sigma). All media were supplemented with 10% fetal bovine serum (EuroClone), 100 IU/ml penicillin (Gibco), and 100 µg/µl streptomycin (Gibco). MDA-MB-231 medium was additionally supplemented with 1% sodium pyruvate (Gibco). Cells were kept at 37°C in a humidified atmosphere containing 5% CO_2_ and 95% air.

Cells were seeded at densities of approximately 1.5–2×10^6^ cells/100-mm, 3×10^5^ cells/60-mm, and 1.5–2×10^5^ cells/35-mm dishes. For siRNA transfections the cell density was halved. Transfections were initiated 24 hours after seeding and were performed with 2 µg plasmid DNA and 2 µl Lipofectamine 2000 (Invitrogen) per ml Optimem I medium (Gibco) according to the supplier's protocol. For siRNA transfections cells were incubated for 48 or 72 h with 40 nM oligonucleotides and 2 µl/ml medium of Lipofectamine 2000 in Optimem. For induction of stress, cells were incubated at 44°C or treated with As_2_O_3_ (Sigma Aldrich) in a humidified atmosphere containing 5% CO_2_ and 95% air. When indicated cells were incubated with 16 nM TPA and 2 µM GF109203X (both from Sigma Aldrich).

### Immunoprecipitation

Cells were treated as indicated in the protocol supplied with the μMACS Epitope-Tagged Protein Isolation Kit (Militenyi Biotec). Briefly, cells were washed twice in ice cold PBS and lysed for 30 minutes on ice in either a lysis buffer supplied with the kit (150 mM NaCl, 1% triton X-100, 50 mM Tris HCl, pH 8.0) (Figs. 1A, 2B, 5, and 7F) or a polysome lysis buffer (100 mM KCl, 5 mM MgCl_2_, 10 mM HEPES, 1 mM DTT, 1% IgePAL CA-630, pH 7.0) (Fig. 1B) for co-precipitation analyses, or in RIPA lysis buffer (10 mM Tris-HCl pH 7.2, 160 mM NaCl, 1% Triton X-100, 1% Na-deoxy-cholate, 0.1% SDS, 1 mM EGTA, 1 mM EDTA) (Figs. 2A, 6, 7A, 7C and 7E) for other experiments. The buffers were supplemented with Complete™ protease inhibitor cocktail without EDTA (Roche). Lysates were cleared by centrifugation at 14,000×*g* for 10 min at 4 °C, and incubated either with anti-GFP-conjugated microbeads for 30 minutes or with 1 µg of antibodies for 1 h to overnight prior to addition of protein G-coupled microbeads and an additional incubation for 30 minutes on rotation at 4 °C. The immune complexes were recovered by applying the lysates on μColumns placed in the magnetic field of a μMACS Separator. Following washes the complexes were eluted with sample buffer.

### Western blot

Proteins were electrophoretically separated by SDS-PAGE and transferred to a PVDF membrane (Millipore). Membranes were pre-incubated with 5% dried milk in PBS followed by incubation with primary antibodies. Membranes were washed, incubated with horseradish peroxidase-labelled secondary antibody, and immunoreactivity was detected with the SuperSignal system (Biological Industries), enhanced chemiluminescence detection system (GE Healthcare) or SuperSignal (Pierce) as substrate. The chemiluminescence was captured with a charge-coupled device camera (Fujifilm) and intensities were quantified with ImageJ.

### GST pull-down

GST/His fusions of G3BP2 variants and G3BP1 were expressed in *Escherichia coli* BL-21(DE3) (Stratagene). Following induction for 4 hours with 1 mM IPTG, bacteria were lysed in buffer (50 mM NaH_2_PO_4_, pH 8, 300 mM NaCl, 10 mM imidazol, 4% Complete™ protease inhibitor cocktail without EDTA) and kept at −80°C overnight. Lysates were sonicated after addition of 1 mM DTT and 1 mg/ml lysozyme. Cleared lysates were mixed with Ni-NTA agarose (Quiagen) for one hour and applied on an Econo-Pac® Disposable Chromatography Columns (BIO-RAD). The agarose was washed twice with washing buffer (20 mM NaH_2_PO_4_, 300 mM NaCl, 250 mM imidazol, 1 mM DTT). His-tagged proteins were eluted in four fractions with 1.5 ml elution buffer each (50 mM NaH_2_PO_4_, 300 mM NaCl, 250 mM imidazol, 1 mM DTT).

GST pull-down assay was performed incubating 80 ng PKCα isozyme (Sigma) with 4 µg of GST-G3BP recombinant proteins in 100 µl binding buffer (20 mM Tris, pH 7.4, 0.1 mM EDTA, 100 mM NaCl, 1 mM DTT) with agitation for 1 hour at 4°C. Thereafter 40 µl μMACS™ anti-GST MicroBeads (Miltenyi Biotec) was added and following 1 hour incubation at 4°C, protein separation was performed on a μ Column in a magnetic field of μMACS Separator (Miltenyi Biotec) according to manufacturers protocol. GST pull-downs were analyzed with SDS-PAGE and Western blotting.

### In vitro kinase assay

Since the GST/His fusion of G3BP2b has the same size as PKCα the GST/His tag was proteolytically removed with thrombin to enable identification on autoradiography. G3BP2b or GST/His fusion of G3BP2 domains (1 µg) was incubated in with 400 ng PKCα (Sigma-Aldrich), 100 µM (2 Ci/mmol) [γ-^32^P]ATP (Perkin Elmer), 20 mM HEPES (pH 7.4) and 10 mM MgCl_2_. Reactions were either supplemented with 0.5 mM EGTA (absence of activators) or with 0.3% Triton X-100, 100 µg/ml phosphatidylserine (Sigma Aldrich), 0.1 mM CaCl_2_ and 20 µg/ml 1,2-diacylglycerol (Avanti). The total volume was 50 µl. Reactions were incubated at 30°C for 20 min and terminated by addition of sample buffer. Samples were separated by SDS-PAGE and subjected to autoradiography and Western blot.

### Immunofluorescence and confocal microscopy

Cells were washed in PBS, fixed with 4% paraformaldehyde in PBS for 4 minutes, washed twice in PBS and thereafter permeabilized and blocked with 5% goat serum or 5% bovine serum albumin and 0.3% Triton X-100 in PBS for 30 minutes. Cells were incubated with primary antibodies for 1 h. Following washes in PBS, cells were incubated with secondary Alexa Fluor 488-, 546-, and/or 633-conjugated antibodies in PBS for 1 h followed by extensive washes in PBS and mounting on object slides using 20 µl PVA-DABCO (9.6% polyvinyl alcohol, 24% glycerol, and 2.5% 1,4-diazabicyclo[2.2.2]octane in 67 mM Tris-HCl, pH 8.0).

For confocal microscopy a Bio-Rad Radiance 2000 confocal system fitted on a Nikon microscope with a 60x/NA 1.40 oil lens or a Zeiss LSM710 was used. Excitation wavelengths were 488 nm (EGFP and Alexa Fluor 488), 543 nm (Alexa Fluor 546), and 637 nm (Alexa Fluor 633) and the emission filters used were HQ515/30 (EGFP and Alexa Fluor 488) and 600LP (Alexa Fluor 546). In triple stainings a HQ600/50 bandpass filter was used for Alexa Fluor 546 detection and a 660LP filter for Alexa Fluor 633. For quantification of stress granules 200 cells were scored for the presence of stress granules, identified either by PABPC1 or G3BP2 antibodies.
